# Ketamine use in a large global sample: Characteristics, patterns of use and emergency medical treatment

**DOI:** 10.1177/02698811241273850

**Published:** 2024-10-17

**Authors:** Karen P Barrios, Dean J Connolly, Jason A Ferris, Larissa J Maier, Monica J Barratt, Adam R Winstock, Cheneal Puljević, Gail Gilchrist

**Affiliations:** 1 National Addiction Center, Institute of Psychiatry, Psychology and Neuroscience, King’s College London, London, UK; 2 Faculty of Public Health and Policy, London School of Hygiene and Tropical Medicine, London, UK; 3 Faculty of Medicine, Center for Health Services Research, The University of Queensland, Brisbane, QLD, Australia; 4 Department of Clinical Pharmacy, University of California, San Francisco, CA, USA; 5 Social Equity Research Center and Digital Ethnography Research Center, RMIT University, Melbourne, VIC, Australia; 6 National Drug and Alcohol Research Center, UNSW Sydney, Sydney, NSW, Australia; 7 Global Drug Survey, London, UK; 8 Institute of Epidemiology and Health Care, University College London, London, UK; 9 Faculty of Medicine, School of Public Health, The University of Queensland, Brisbane, QLD, Australia

**Keywords:** Emergency medical treatment, Global Drug Survey, ketamine, risk factors, substance use

## Abstract

**Background:**

Ketamine’s popularity has surged globally in the past decade, especially among young men. Emergency department visits due to its toxicity remain relatively rare, often linked to co-occurring use of other substances.

**Aims:**

Using data from the Global Drug Survey (GDS) 2018, this study explored the correlates associated with lifetime and past-year ketamine use, and estimated the socio-demographic characteristics, usage patterns and experiences of respondents seeking emergency medical treatment (EMT) after ketamine use.

**Methods:**

Secondary analysis of GDS 2018, an online cross-sectional survey on drug use patterns conducted between November 2017 and January 2018.

**Results:**

The survey received 130,761 valid responses, with 5.93% reporting lifetime ketamine use, of which 57.70% used ketamine within the past year. Predominantly, respondents were from Germany, England and Denmark. Within the past year, 8.55% met the criteria for ketamine dependence. Respondents who used ketamine in their lifetime tended to be young (mean (x̄) = 27.37 years), men, heterosexual and of white ethnicity. Younger age (x̄ = 24.84 years), gay sexual orientation, student status, past-year use of other drugs and no lifetime mental health diagnosis were associated with past-year ketamine use. Among 4477 respondents reporting past-year ketamine use, 120 adverse events were reported, with less than 0.10% prompting EMT seeking.

**Conclusion:**

The study reveals frequent ketamine use but low harm occurrence, underscoring the complex interplay between ketamine use, substance use and dependence, and related factors. This underscores the need to reassess EMT priorities, implement tailored harm reduction strategies and incorporate comprehensive screening for addressing ketamine and substance dependence challenges.

## Introduction

### History

Ketamine, initially developed in the 1960s as an alternative to phencyclidine, is a synthetic dissociative anaesthetic and analgesic (Li and Vlisides, 2016; Oduntan and Gool, 1970) widely used in both human and veterinary medicine (Beck, 1976; Morgan and Curran, 2012) due to its ability to induce anaesthesia while preserving cardiovascular stability (Bettschart-Wolfensberger and Larenza, 2007; Miranda-Cortés et al., 2020). In recent decades, it has been investigated as a treatment for major depressive disorder and other mental health conditions (Matveychuk et al., 2020), with randomised trials showing rapid antidepressant effects (Berman et al., 2000; Matveychuk et al., 2020; Murrough et al., 2013). Specifically, in the treatment of treatment-resistant depression (TRD), there has been a growing interest in the use of ketamine (e.g. intranasal or intravenous; McIntyre et al., 2021). Research indicates that ketamine’s anti-depressant effects are brought about by enhancing synaptic plasticity and functional connectivity (Meshkat et al., 2023). It reduces symptoms such as low mood, suicidal thoughts and anhedonia (Jawad et al., 2023; Phillips et al., 2019; Rodrigues et al., 2022). Moreover, even the most treatment-resistant patients (i.e. those who have failed to respond to at least two prior pharmacotherapies) have shown potential benefits from ketamine (Alnefeesi et al., 2022). The effectiveness of ketamine has been sustained over extended treatment periods, as confirmed by a systematic review that included regression analyses, demonstrating that the therapeutic effects of ketamine did not significantly decline over time or with repeated dosing (Alnefeesi et al., 2022).

As the therapeutic potential of ketamine was identified in medical settings (Dotson et al., 1995), ketamine became popular among ‘underground’ party attendees in the 1970s (Siegel, 1978). Large-scale techno parties, usually called ‘raves’, originated in the United States (US) and gained mass popularity in the United Kingdom (UK) and other countries during the 1980s (Dotson et al., 1995; Ter Bogt and Engels, 2005). These events became common environments for ketamine and other drug use, with the substance attaining prominence as a ‘club drug’ (Dotson et al., 1995; Sassano-Higgins et al., 2016), commonly used in ‘mainstream’ commercialised nightlife internationally (Le Dare et al., 2021). Furthermore, its sexualised use is included in some definitions of chemsex, which appears to be increasingly prevalent in communities of men who have sex with men (MSM; Cohen et al., 2004; Maxwell et al., 2019). Some individuals also self-medicate with ketamine (Bennadi, 2014), often involving micro-dosing (Anderson et al., 2019; Kuypers et al., 2019), to improve well-being and enhance cognitive and emotional processes (Chaves et al., 2020; Kuypers et al., 2019).

### Effects

Ketamine has a wide therapeutic window and is typically considered less risky than other drugs (e.g. alcohol, heroin, cocaine; Nutt et al., 2010), it is associated with adverse psychedelic, cognitive and physical effects, which seem to vary by dose and frequency (Palamar et al., 2022). At lower doses, ranging from 0.1 to 0.6 mg/kg (Li and Vlisides, 2016), it induces mild relaxation, dissociation and euphoria, distorting time, space and body perception (Morgan and Curran, 2012; Pal et al., 2002; Wolff and Winstock, 2006). As the dose increases, it can lead to a more profound state known as the ‘K-hole’ (Morgan and Curran, 2012), which can have varying effects on individuals (Stirling and McCoy, 2010). Higher and more frequent doses impair neuropsychological function in the short and long term (Curran and Monaghan, 2001; Krystal et al., 1994; Morgan et al., 2010), including working, episodic and procedural memory, and verbal learning (Morgan et al., 2004; Zhornitsky et al., 2022). Additionally, it can lead to increased depressive symptoms (Kalsi et al., 2011; Morgan et al., 2010), and various health issues including cardiovascular effects (Kalsi et al., 2011; Ward and Standage, 2003), K-cramps, vomiting, dysphagia (associated with gastro-intestinal toxicity; Kalsi et al., 2011; Liu et al., 2017) and urological disorders, such as haemorrhagic cystitis, hydronephrosis, contracted bladder, ureteral stenosis or bladder fibrosis – damage which in severe cases may be irreversible (Castellani et al., 2020; Chen et al., 2011; Tsai et al., 2009; Winstock et al., 2012).

### Prevalence

Over the past decade, global illicit ketamine use has increased (Hottat and Hantson, 2023). For instance, a comprehensive study analysing wastewater from over 100 European cities in 2022 revealed heightened availability and utilisation of ketamine, with the highest mass loads found in municipal wastewater in cities across Denmark, Spain, Italy and Portugal (European Monitoring Centre for Drugs and Drug Addiction, 2022). Moreover, an increase in people seeking treatment for ketamine-related issues was observed in Belgium, Spain, France and Italy in 2021, pointing towards an emerging trend of ketamine use in these countries (European Monitoring Centre for Drugs and Drug Addiction, 2023). This trend was reported by other wastewater analyses in various nations, including Italy, the UK and the US (Adhikari et al., 2023; Bishop et al., 2020; Castiglioni et al., 2015; Du et al., 2015; Rice et al., 2020), supporting its prevalent use in festival and party scenes (Da Cunha et al., 2021). Moreover, ketamine seizures also increased in Europe, from 91,737 kg in 2011 to 840,482 kg in 2021 (European Monitoring Centre for Drugs and Drug Addiction, 2023).

In Australia, between December 2022 and April 2023, ketamine excretion levels rose significantly in both capital city and regional sites, reaching record highs (Australian Criminal Intelligence Commission, 2023). In England and Wales, adult last-year ketamine use increased from 0.5% in 2010 to 0.9% in 2022, with a notable rise among 16- to 24-year-olds, from 1.7% in 2010 to 3.2% in 2020 (Office for National Statistics, 2020, 2022). New ketamine-related treatment cases tripled since 2014–2015, and in 2021–2022, 0.8% of ‘club drug’ treatment cases addressed ketamine issues (Office for Health Improvement and Disparities, 2023a). Individuals less than 18 years of age seeking ketamine treatment rose from under 1% in 2015–2016 to 5% in 2020–2021 and continuing in 2022 (Office for Health Improvement and Disparities, 2023b).

Ketamine dependence, characterised by cravings and tolerance (American Psychological Association, 2013; Pal et al., 2002), is associated with prolonged and heavy use (Van Amsterdam and Van Den Brink, 2022) and has been observed in various populations, including healthcare providers (Goyal et al., 2014; Hurt and Ritchie, 1994; Moore and Bostwick, 1999), people with different ethnic backgrounds (e.g. Asian, Latino, White; Amaro et al., 2010; Lim, 2003) and those with co-occurring conditions (Błachut et al., 2009).

### Who uses ketamine?

The surge in recreational ketamine use has led to studies examining usage patterns and profiles of people who use ketamine. In Spain, participants were mainly middle-class men with an average age of 26.2 years (Vidal Giné et al., 2016). In China, the majority were men aged 21–30 years, single and unemployed (Tang et al., 2022). In Taiwan, the mean age was 24.4 years, with participants being predominantly men, employed and heterosexual (Lee et al., 2022). In New York City, ketamine use increased among dance music party attendees (from 5.9% in 2016 to 15.3% in 2019), predominantly men, white, heterosexual and with a mean age of 25.3 years (Palamar and Keyes, 2020).

Additionally, research has delved into ketamine-related deaths, revealing their relative infrequency compared to fatalities associated with many other drugs. Studies in England and Australia showed that, in most cases, these deaths mostly occurred among employed men living with others (Corkery et al., 2021; Darke et al., 2021). The primary cause of these ketamine-related deaths was identified as drug toxicity, with polydrug use being common (Corkery et al., 2021; Darke et al., 2021), and other research shows concurrent use of ketamine with drugs like alcohol, GHB/GBL (γ-hydroxybutyric acid/γ-butyrolactone), cocaine, and MDMA (3,4-methylenedioxymethamphetamine; Wood et al., 2008). Additionally, post-mortem findings frequently indicate the presence of ketamine alongside other substances or in trauma cases, suggesting that fatal ketamine overdoses typically result from polysubstance use (Kalsi et al., 2011). While ketamine-related deaths remain relatively infrequent, the broader statistics concerning other drugs have shown increasing rates of death in comparison. For instance, in the US alone in 2021, there were 106,699 drug overdose deaths, with the majority attributed to opioids. Opioids were implicated in 80,411 overdose deaths, accounting for 75.4% of all drug overdose deaths in 2021, increasing from 68,630 deaths in 2020 (Centres for Disease Control and Prevention, 2021; National Institute on Drug Abuse, 2023).

### Ketamine and emergency medical treatment

Finally, some studies have investigated the link between ketamine consumption and emergency department (ED) presentations. In Hong Kong, individuals who use ketamine tend to have higher ED attendance rates compared to general ED patients, often due to issues like gastrointestinal or lower urinary tract symptoms, highlighting acute adverse effects (Chu et al., 2007; Lee et al., 2011; Ng et al., 2010; Wei et al., 2021). A 2021 cohort study in Hong Kong found that ketamine use significantly increased ED attendance odds compared to opioids, but was seldom associated with deaths (Wei et al., 2021). In Taiwan, one study revealed ketamine’s popularity among young adults, but only identified two people attending the ED, suggesting that ketamine may not frequently lead to ED visits and showing that legal concerns are a potential barrier to seeking EMT, similar to people who use opioids (Chun-Jen Chen et al., 2012). Another study, focused on ED visits due to substance-related complications in Taipei, found that among the cases linked to drug use, ketamine was detected in 21.7% of instances and was particularly prevalent among individuals aged 11–20 years (Weng et al., 2020). Reports also indicate that attendees to the ED who use ketamine are typically men, around 25 years old, mostly Caucasian and often report various neurological, gastrointestinal, urological, and cardiac symptoms and anxiety (Pavarin et al., 2019; Weiner et al., 2000).

Given the extensive history, diverse effects and increasing prevalence of ketamine use, this secondary analysis of data from a large and diverse multi-country sample of respondents from the Global Drug Survey (GDS) 2018 aimed to:

Explore and analyse the correlates associated with the use of ketamine by people who have used it in their lifetime and during the past year.
Describe ketamine use patterns, and characteristics of those seeking and not seeking EMT for ketamine-related issues.
Calculate the per-event risk of past-year ketamine intake events that led to EMT seeking.

## Methods

### Design

A secondary analysis was conducted using data from the world’s largest annual anonymous online cross-sectional survey, GDS. Data collection was conducted between 6th November 2017 and 10th January 2018. The survey collects self-reported data on drug use and behaviours from a diverse global sample via a secure website (Barratt et al., 2017; Winstock et al., 2022). The respondents were recruited using purposive sampling, a non-probabilistic technique (Barratt et al., 2017).

The online survey was accessible to participants through the GDS website and promoted on social media, online drug forums and by several partner organisations (Winstock et al., 2022). Prior to participating in the survey, all participants confirmed that they were aged 16 years or older, had used at least one drug (including alcohol or tobacco) in the past 12 months and provided online consent. To ensure their anonymity, no IP addresses or other identifying information was collected from respondents, as the information collected was sensitive (Barratt et al., 2017). Further details on the GDS’s methods are provided elsewhere (Barratt et al., 2017; Winstock et al., 2022).

Ethical approval was received from University College London (11671/001: GDS), The University of New South Wales (HREC HC17769) and The University of Queensland (No: 2017001452) Research Ethics Committees.

### Measures

Participant characteristics were collected via self-report: socio-demographic data included age, gender identity, sexual orientation, ethnicity, country of residence, employment and student status. The terms ‘male’ and ‘female’ were used to capture responses pertaining to gender. To ensure clarity in subsequent discussions, when referring to survey responses categorised as ‘male’ and ‘female’, the terms ‘men’ and ‘women’ will be used, respectively. This distinction is made to align our terminology with a gender-focused perspective, acknowledging that the original labels often refer to sex, a separate construct or might have implied a binary understanding of gender. Participants were asked about their history of drug use (never, in the last 30 days, between 31 days and 12 months ago, more than 12 months ago) from an extensive list of substances that included ketamine. Participants indicating a history of use with a drug were then redirected to answer detailed questions about the use of these specific substances. For the analyses of this study, the variables related to ketamine use were used. Participants were asked if they had sought EMT following ketamine use and subsequent questions characterised these incidents. They were asked about their desire to reduce drug use, whether they had ever been diagnosed with a mental illness and to select from a list of specific mental health diagnoses in case they were. Supplemental Table 1 provides a list of questions and corresponding response options pertaining to all variables incorporated in the article.

Participants were asked about their use of other drugs in the past year. Variables related to drug use in the past year were grouped to include them in the bivariable and multivariable analyses. This categorisation aimed to consolidate related drug types into broader categories, reducing the potential for multicollinearity. Supplemental Table 2 presents the drug variables used in the analysis and their corresponding new categorisation to use in the analysis.

The number of adverse events was determined by summing instances where EMT was actively sought with occurrences where respondents thought EMT should have been sought but refrained. Additionally, the Severity of Dependence Scale (SDS) for multiple drugs was used. In this scale, five items are provided regarding impaired control over drug taking and preoccupation and anxieties about drug use (Gossop et al., 1995). The results of each item were summed to provide a dimensional score in the range of 0–15, where higher scores exceeding a threshold indicate more severe dependence (Gossop et al., 1995). It is worth noting that SDS scores can suggest probable dependence. For brevity, the term ‘dependence’ will be used throughout the rest of the paper. The SDS cut-off scores were as follows: ketamine, MDMA and cocaine had a cut-off of three (Bruno et al., 2011; Fernández-Calderón et al., 2016; Kaye and Darke, 2002; Tung et al., 2014), cannabis and amphetamines had a cut-off of four (Topp and Mattick, 1997; Van der Pol et al., 2013), and GHB/GBL had a cut-off of five (Degenhardt et al., 2002).

### Data analysis

The present analytic sample included participants who completed the ketamine use module and provided valid responses to any of the demographic questions located at the end of the survey. These demographic questions are denoted with an asterisk (*) in Supplemental Table 1. The statistical analysis was conducted using SPSS version 29 (IBM Corp., 2023). Counts, percentages, mean, median, interquartile range (IQR) and standard deviation (SD) were used to summarise the demographic characteristics of the total sample of respondents with a lifetime history of ketamine use, followed by participants who used ketamine in the past year, and those who sought or did not seek EMT. Valid percentages rather than absolute percentages were reported when data were missing. A bivariable analysis using binary logistic regression was employed to examine the associations between demographic and drug use variables, and respondents who reported ketamine use within the last year or reported not using in the same period. Odds ratios (OR) and their corresponding 95% confidence intervals (CI) were calculated using bivariable analysis to quantify the relationships between each variable and the odds of ketamine use in the past year. Applying hierarchical model-building approaches (Hosmer and Lemeshow, 2013), variables related to ketamine use (*p* < 0.1), as identified through bivariable analyses, were included in subsequent multivariable analysis using a backward stepwise multiple logistic regression model. Checks for multicollinearity among the independent variables were performed to ensure the validity of the results.

Additionally, as reported by Kopra et al. (2022a, 2022b), the per-event risk of seeking EMT was determined by dividing the number of participants who reported seeking EMT for ketamine use within the past year by the estimated total times ketamine was used among those who used it in the past year.

$$\mathrm{Per}-\mathrm{event}\;\mathrm{risk}=\;\;\frac{N\;\mathrm{participants}\;\mathrm{reporting}\;\mathrm{EMT}}{\begin{array}{l} \mathrm{Mean}\;\mathrm{times}\;\mathrm{used}\;\mathrm{past}\;\mathrm{year}\;\times \\ N\;\mathrm{past}\;\mathrm{year}\;\mathrm{users} \end{array}}$$

## Results

A total of 130,761 respondents took part in GDS 2018, of which 7759 (5.93%) reported lifetime ketamine use. Of these, 4477 (57.70%) reported using ketamine within the past year.

### Lifetime ketamine use

Table 1 shows the demographic profile of respondents who have reported using ketamine in their lifetime (*N* = 7759). The mean age of the sample was 27.37 years; the majority identified as men (69.44%), heterosexual (76.22%) and Caucasian (90.63%). Participants reported the highest representation from Germany (32.08%), followed by England (10.41%), Denmark (7.62%) and the US (6.33%). Most respondents reported being in paid employment (66.24%) and not currently studying (60.67%). Among respondents with a lifetime mental health diagnosis (*n* = 2352), the most frequently reported conditions were depression and anxiety (Table 1).

**Table 1.** Demographics of Global Drug Survey 2018 respondents who reported lifetime ketamine use (*N* = 7759).

Variable	*N* (%)
Age	
Mean (SD^a^)	27.37 (8.21)
Median (IQR^b^)	25.00 (31, 21)
Gender	
Men	5388 (69.44)
Non-binary/different identity	122 (1.57)
Women	2249 (28.99)
Sexual orientation	
Bisexual	1090 (14.96)
Heterosexual	5554 (76.22)
Homosexual	460 (6.31)
Gay	404 (5.54)
Lesbian	56 (0.77)
Other	183 (2.51)
Missing	(472)
Ethnicity	
Aboriginal/Maori	9 (0.12)
Asian	32 (0.44)
Black African/Black Caribbean	18 (0.25)
Black American	1 (0.01)
Hispanic/Latino	128 (1.75)
Mixed	296 (4.04)
Native American	9 (0.12)
South East Asian	36 (0.49)
White	6647 (90.63)
Other	158 (2.15)
Missing	(425)
Country of residence^c^	
Australia	370 (4.77)
Austria	258 (3.33)
Denmark	591 (7.62)
England	808 (10.41)
Germany	2489 (32.08)
Italy	200 (2.58)
Netherlands	437 (5.63)
Scotland	223 (2.87)
Switzerland	211 (2.72)
United States	491 (6.33)
Other	1681 (21.67)
Employment status	
Paid employment	4767 (66.24)
Unemployed	2430 (33.76)
Missing	(562)
Education	
Currently studying	2904 (39.33)
Not currently studying	4480 (60.67)
Missing	(375)
Mental health diagnosis^d^	
No	5391 (69.62)
Yes	2352 (30.38)
Missing	(16)
Which mental health diagnosis^e^	
ADHD^f^	405 (17.36)
Anxiety	1109 (47.54)
Bipolar affective disorder	256 (10.97)
Depression	1588 (68.07)
Psychosis	164 (7.03)
Other	480 (20.57)
Missing	(5426)

a Standard deviation.

b Interquartile range.

c The frequencies of the countries with the highest responses are displayed, while the remaining countries are grouped under the category ‘other’.

d Lifetime mental health diagnosis.

e Participants could provide ⩾1 response; hence, the total count and percentage do not add up to 2352 and 100%, respectively.

f Attention deficit hyperactivity disorder.

### Past-year ketamine use

Table 2 shows the patterns of ketamine use among respondents who used ketamine in the last year. Among them, most reported using ketamine in the past month, primarily through insufflation (i.e. snorting; 92.08%). Drinking alcohol concurrently with the use of ketamine was common (45.99%), and the majority of respondents also reported the use of other drugs in the past 12 months (i.e. cannabis, stimulants and tobacco, among others; Table 2). Additionally, among respondents who used ketamine in the past year (*n* = 4477), SDS scores for ketamine and other drugs were calculated, showing varying levels of drug dependence. Noting that a person may have reported concerning SDS scores for more than one drug, results show that the drug the greatest number of participants reported probably dependence on was cannabis (25.81%), followed by cocaine (19.14%), amphetamine powder, amphetamine paste, or methamphetamine (17.18%), MDMA (16.11%), ketamine (8.55%) and GHB/GBL (5.73%). The SDS scores of the six drugs mentioned above were summed to assess the number of drugs the respondents were dependent on. Of the 4457 respondents who responded to those questions, most were not dependent on any drug (*n* = 2321, 52.08%). Additionally, 1469 respondents were dependent on one drug (32.96%), 535 were dependent on two drugs (12.00%), 131 respondents were dependent on three or more drugs (2.93%) and just one person reported being dependent on six drugs.

**Table 2.** Patterns of drug use by Global Drug Survey 2018 respondents who used ketamine in the last year (*N* = 4477).

Characteristics of drug use	*N* (%)
Past month ketamine use	
No	2623 (58.59)
Yes	1854 (41.41)
Most common route of administration	
Inject	78 (1.82)
Oral	214 (5.00)
Rectal	18 (0.42)
Smoke	9 (0.21)
Snort	3939 (92.08)
Other	20 (0.47)
Missing	(199)
Typical amount consumed in a session of use (grams)	
Median (IQR^a^)	0.30 (0.50, 0.20)
⩽1	3770 (86.67)
>1	88 (2.02)
Don’t know	492 (11.31)
Missing	(127)
Ketamine source	
Friends	745 (29.46)
Friends of friends	397 (15.70)
Dealers that they know	667 (26.37)
On the street/festival/club	167 (6.60)
Shopfronts	7 (0.28)
Darknet markets (purchased by self)	313 (12.38)
Darknet markets (purchased by others)	66 (2.61)
Open websites	36 (1.42)
WhatsApp	27 (1.07)
Other social media apps	17 (0.67)
Another source	87 (3.44)
Missing	(8)
Past-year alcohol consumption	
No	119 (2.67)
Yes	4338 (97.33)
Missing	(20)
Concurrent ketamine and alcohol use^b^	
Never (0%)	1499 (34.41)
Rarely (25%)	854 (19.61)
Sometimes (50%)	506 (11.62)
Often (75%)	499 (11.46)
Always (100%)	998 (22.91)
Missing	(121)
Other drug use (past year)^c^	
Cannabinoids	4033 (90.08)
Inhalants	983 (21.96)
NPS^d^	905 (20.21)
Opioids	359 (8.02)
Psychedelics	2638 (58.92)
Stimulants	4311 (96.29)
Tobacco	3689 (82.40)

a Interquartile range.

b Percentages represent how much of the time people use ketamine while using alcohol.

c Participants could provide ⩾1 response; hence, the total count and percentage do not add up to 4477 and 100%, respectively.

d Novel psychoactive substances.

### Lifetime and past-year ketamine use

Of all respondents who indicated ever using ketamine, Table 3 shows the results of the comparison of demographic and drug use characteristics between respondents who reported using ketamine in the past 12 months (*n* = 4477) and those who did not use ketamine during the same period (*n* = 3282). After performing the bivariable analysis, the following variables were included in the multivariable analysis: age, sexual orientation, employment status, education, mental health diagnosis and other drug use in the past year.

**Table 3.** Comparison of demographic profile and drug use between Global Drug Survey 2018 respondents who have used ketamine in their lifetime and who used and did not use ketamine in the past 12 months.

	Ketamine use in the past 12 months					
	Yes	No	Bivariable		Multivariable	
	(*N* = 4477)	(*N* = 3282)				
	*N* (%)	*N* (%)	OR^a^ (95% CI^b^)	*p*	OR (95% CI)	*p*
Age						
Mean (SD^c^)	24.84 (6.68)	30.83 (8.81)	0.90 (0.89–0.91)	**<0.001**	0.91 (0.91–0.92)	**<0.001**
Gender						
Men	3131 (69.94)	2257 (68.77)	1	–	–	–
Non-binary/different identity	74 (1.65)	48 (1.46)	1.11 (0.77–1.60)	0.57	–	–
Women	1272 (28.41)	977 (29.77)	0.94 (0.85–1.04)	0.21	–	–
Sexual orientation						
Heterosexual	3159 (74.73)	2395 (78.27)	1	–		
Gay	248 (5.87)	156 (5.10)	1.21 (0.98–1.48)	0.08	1.62 (1.23–2.14)	**<0.001**
Lesbian	34 (0.80)	22 (0.72)	1.17 (0.68–2.01)	0.56	1.51 (0.77–2.99)	0.23
Bisexual	683 (16.16)	407 (13.30)	1.27 (1.11–1.45)	**<0.001**	1.02 (0.86–1.20)	0.85
Other	103 (2.44)	80 (2.61)	0.98 (0.73–1.31)	0.87	0.88 (0.60–1.24)	0.42
Missing	(250)	(222)				
Employment status						
Paid employment	2593 (62.26)	2174 (71.70)	1	–	–	–
Unemployed	1572 (37.74)	858 (28.30)	1.54 (1.39–1.70)	**<0.001**	–	–
Missing	(312)	(250)				
Education						
Not currently studying	2285 (53.51)	2195 (70.67)	1	–	–	–
Currently studying	1993 (46.59)	911 (29.33)	2.10 (1.91–2.32)	**<0.001**	1.26 (1.11–1.42)	**<0.001**
Missing	(199)	(176)				
Mental health diagnosis^d^						
Yes	1263 (28.27)	1089 (33.24)	1	–	–	–
No	3204 (71.73)	2187 (66.76)	1.26 (1.15–1.39)	**<0.001**	1.21 (1.07–1.38)	**0.003**
Missing	(10)	(6)				
Which mental health diagnosis^e^						
ADHD^f^	209 (16.65)	196 (18.18)	0.90 (0.73–1.11)	0.33	–	–
Anxiety	600 (47.81)	509 (47.22)	1.02 (0.87–1.21)	0.78	–	–
Bipolar affective disorder	129 (10.28)	127 (11.78)	0.86 (0.66–1.11)	0.25	–	–
Depression	851 (67.81)	737 (68.37)	0.97 (0.82–1.16)	0.77	–	–
Psychosis	74 (5.90)	90 (8.35)	0.69 (0.50–0.95)	**0.02**	–	–
Other	269 (21.43)	211 (19.57)	1.12 (0.92–1.37)	0.27	–	–
Other drug use past year^g^						
Alcohol	4338 (97.33)	3030 (92.98)	2.60 (2.10–3.21)	**<0.001**	1.69 (1.27–2.24)	**<0.001**
Cannabinoids	4033 (90.08)	2595 (79.07)	2.40 (2.11–2.74)	**<0.001**	–	–
Inhalants	983 (21.96)	270 (8.23)	3.14 (2.72–3.62)	**<0.001**	2.50 (2.10–2.99)	**<0.001**
NPS^h^	905 (20.21)	286 (8.71)	2.65 (2.30–3.06)	**<0.001**	1.59 (1.34–1.90)	**<0.001**
Opioids	359 (8.02)	169 (5.15)	1.61 (1.33–1.94)	**<0.001**	1.22 (0.96–1.55)	0.10
Psychedelics	2638 (58.92)	952 (29.01)	3.51 (3.19–3.86)	**<0.001**	2.34 (2.09–2.63)	**<0.001**
Stimulants	4311 (96.29)	2253 (68.65)	11.86 (9.99–14.08)	**<0.001**	6.51 (5.36–7-92)	**<0.001**
Tobacco	3689 (82.40)	2492 (75.93)	1.48 (1.33–1.66)	**<0.001**	0.84 (0.73–0.98)	**0.02**
Multivariable model statistics						
Hosmer and Lemeshow test (X2)					9.86	0.27
Model classification						74.15

a Odds ratio.

b Confidence interval.

c Standard deviation.

d Lifetime mental health diagnosis.

e People who responded they had a lifetime mental health diagnosis (1263 and 2352, respectively, for people who use and did not use ketamine in the past 12 months) could respond which one. Participants could provide ⩾1 response; hence, the total number and percentage do not add up to 100%.

f Attention deficit hyperactivity disorder.

g Participants could provide ⩾1 response; hence, the total count does not add up to 4477 and 3282, respectively.

h Novel psychoactive substances.

**Bold** text indicates statistical significance (*p*<0.05).

The mean age of respondents who used ketamine in the past 12 months (24.84 years) was lower than the mean age of respondents who had not used it in the past 12 months (30.83 years). The bivariable analysis showed a statistically significant association between age and ketamine use, and this association remained significant in the multivariable analysis (Table 3). In the bivariable analysis, sexual orientation was significantly associated with ketamine use (*p* = 0.005), with bisexual respondents being significantly more likely than heterosexual respondents to report ketamine use in the last 12 months. In the multivariable analysis, sexual orientation was significantly associated with ketamine use in the past 12 months (*p* = 0.01), and gay men had higher odds of ketamine use compared to heterosexual respondents (Table 3).

In the bivariable analysis, respondents who were currently studying had higher odds of using ketamine in the last 12 months compared to those who were not studying. Similarly, respondents who were currently unemployed had higher odds of ketamine use than those in paid employment. However, in the multivariable analysis, only education remained significantly associated with ketamine use, with current students having 1.26 times the odds of reporting ketamine use compared to non-students (Table 3). Additionally, respondents without a lifetime mental health diagnosis had 1.26 times the odds of using ketamine in the last 12 months, and the association remained significant in the multivariable analysis (Table 3). Although psychosis was identified as significant in the bivariable analysis, it was not included in the multivariable analysis due to already incorporating a broad lifetime mental health diagnosis variable, including psychosis would reduce sample size, compromising statistical robustness.

Finally, in the bivariable analysis, respondents who reported ketamine use in the last 12 months had higher odds of reporting using various other drugs in the past year compared to respondents who did not use ketamine in the past 12 months, including alcohol, cannabinoids, inhalants, new psychoactive substances (NPS), opioids, psychedelics, stimulants and tobacco. In the multivariable analysis, some of these associations remained (alcohol, inhalants, NPS, psychedelics, stimulants and tobacco; Table 3). The most striking association observed was the significantly higher odds of using ketamine among respondents who reported using stimulants. Specifically, the multivariable analysis revealed that respondents using stimulants had 6.51 times greater odds of using ketamine in the past 12 months compared to respondents who had used ketamine but not in the last 12 months (Table 3).

In summary, the results indicate that younger age, gay sexual orientation, currently studying and not having a lifetime mental health diagnosis were associated with higher odds of past-year ketamine use. Additionally, respondents who used ketamine in the past year had higher odds of reporting the use of other drugs in the past year (Table 3).

### Help seeking for ketamine use

**Figure 1.**
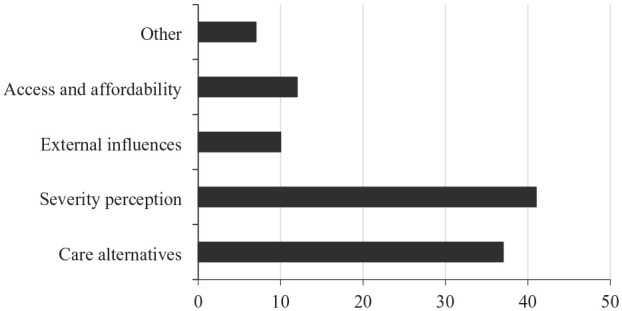
Reasons for not seeking emergency medical treatment.

Among the 4933 participants who answered the EMT question, 29 respondents (0.58%) reported seeking EMT following past-year ketamine use. There was no significant difference in age between respondents who sought EMT (x̄ = 24.70 years, SD = 6.67) and those who did not (x̄ = 24.90 years, SD = 5.76). The percentage of women (34.48%) was higher in the group who sought EMT compared with those who did not (28.02%). Most respondents in both groups were heterosexual, followed by bisexual (16.20% in the group who did not seek EMT and 25.00% in the group who sought EMT). Most respondents who sought EMT were unemployed (54.17%) and not studying (56.00%) while those who did not seek EMT were mostly in paid employment (62.34%). Most respondents who sought EMT reported ever having received a mental health diagnosis (69.23%) while those who did not seek EMT reported not having one (71.88%). Figure 1 shows the reasons behind respondents’ decisions not to seek EMT within the past 12 months.

Table 4 shows the results regarding EMT seeking and desire to reduce ketamine consumption among respondents who did and did not seek EMT in the past 12 months. The majority in the EMT-seeking group reported wanting to reduce their ketamine intake, whereas the majority who did not seek EMT expressed a preference for not reducing their ketamine usage. Additionally, most respondents who sought EMT reported seeking this only on a single occasion, and in both groups most respondents did not think of seeking EMT in the past 12 months.

**Table 4.** Comparison of desires to reduce ketamine usage and emergency medical treatment (EMT) seeking behaviour in the past year.

Variables related to EMT-seeking and ketamine use reduction	Did not seek EMT in past 12 months (*N* = 4904)	Sought EMT in past 12 months (*N* = 29)
	*N* (%)	*N* (%)
Would you like to use less ketamine over the next 12 months?		
No	3881 (79.84)	13 (44.83)
Yes	980 (20.16)	16 (55.17)
Missing	(43)	(0)
Would you like help to use less ketamine over the next 12 months?		
No	530 (95.15)	5 (50.00)
Yes	27 (4.85)	5 (50.00)
Missing	(423)	(6)
Which one would be most likely to use to help?		
Alternative therapy	5 (21.74)	1 (25.00)
Counselling at general practitioner	3 (13.04)	–
Counselling via email	3 (13.04)	–
Self-help tool	6 (26.09)	–
Therapy at a specialist drug service	6 (26.09)	3 (75.00)
Missing	(4)	(6)
Times EMT sought in the past 12 months		
1	–	22 (78.57)
2	–	5 (17.86)
4	–	1 (3.57)
Missing	–	(1)
Thought of seeking EMT in the past 12 months		
No	4787 (97.87)	25 (86.21)
Yes	104 (2.13)	4 (13.79)
Missing	(13)	(0)
Times thought should seek EMT in the past 12 months		
1	53 (76.81)	1 (33.33)
2	13 (18.84)	1 (33.33)
3	2 (2.90)	1 (33.33)
5	1 (1.45)	–
Missing	(35)	(1)

Among those who responded to the EMT question, the mean number of times ketamine was used in the past year was 12.04, amounting to an estimated 59,393.32 number of total times ketamine was used. The calculation of the per-event risk estimate yielded 0.00048, indicating that approximately 0.05% of past-year ketamine intake events in this specific sample led to respondents seeking EMT services. In other words, approximately one out of every 2000 times ketamine was used in the past year leading to seeking EMT.

Additionally, there were 120 adverse events, with most respondents reporting a single event (*n* = 70). Fifteen respondents reported two events, two reported three events, one reported four events and another two reported five events. Of these adverse events, 28.89%, or approximately three in ten, resulted in respondents seeking EMT.

## Discussion

The analysis revealed varying levels of ketamine and other drug use and dependence. While comprehensive large-scale studies illustrating the incidence of ketamine dependence are lacking, the existing literature does provide evidence of its presence, primarily within specific populations such as medical professionals and people with co-occurring mental health conditions (Błachut et al., 2009; Goyal et al., 2014; Hurt and Ritchie, 1994; Moore and Bostwick, 1999). Findings from a UK study show that 56.7% of people who use ketamine frequently, 43.3% of people who use it infrequently and 60% of people who stopped using it expressed concerns about addiction/dependence, actively searching for the drug, or the perception that ketamine had become a significant component of their social interactions (Muetzelfeldt et al., 2008). The findings of this study align with the available literature, as probable ketamine dependence was identified in 357 (8.55%) people. However, it is noteworthy that among the participants who reported probable ketamine dependence, 132 (86.84%) reported they would not like help to use less, which is consistent with the results reported in other GDS samples (2009–2010), in which only 5.9% (*n* = 47) of respondents who use ketamine in the past month indicated that they would like help with their ketamine use (Winstock et al., 2012).

While the therapeutic potential of ketamine, particularly in TRD, is promising, it is important to consider its potential risks. Literature suggests that since ketamine is commonly used recreationally and can lead to dependence (also identified in our study), using oral or intranasal ketamine formulations to treat depression might heighten the risk of developing dependence over time, even with lower doses being used (Kolar, 2018; Marcantoni et al., 2020). Limited clinical research suggests that administrations of ketamine in professionally supervised environments have not led to dependence among patients with TRD (Kolar, 2018; Marcantoni et al., 2020). However, these studies often lack comprehensive assessments of dependence risk (Le et al., 2022). Therefore, ongoing vigilance and further research are necessary to fully understand and mitigate the risks, especially when considering ketamine for long-term use in depression treatment (Sheng et al., 2016).

This study shows that ketamine use in the past 12 months is significantly associated with younger age at both the bivariable and multivariable levels. Specifically, it demonstrates that individuals who did not use ketamine in the past year are significantly older than those who did. This finding aligns with existing research on recreational drug use, which typically shows a higher prevalence of drug use, including ketamine, among younger adults, and a decline in its use after young adulthood (Bachman et al., 2014; NIDA, 2020). Research has identified several factors contributing to this trend. Younger individuals, particularly those in their late 10s and early 20s, typically use more recreational drugs associated with their greater levels of risk-taking behaviour and attendance at social environments that promote such activities, such as clubs and festivals (EMCDDA, 2022; Winters and Arria, 2011). As individuals transition into later stages of adulthood, they often assume increased responsibilities, such as careers, families and financial obligations, which may serve as deterrents to drug use (Bachman et al., 2014).

This study shows that gay men had 1.62-fold greater odds of using ketamine within the past year compared to their heterosexual counterparts. This finding holds particular significance in light of broader contextual understandings of substance use patterns. Previous studies have demonstrated that the use of specific substances, including ketamine and stimulants, is linked with chemsex within communities of MSM (Amundsen et al., 2023). Chemsex is often associated with condomless sex and poly-drug use, conferring significant risk to participants (González-Baeza et al., 2018; Pufall et al., 2018; Soria, 2021). A growing body of evidence spanning multiple studies underscores the connection between chemsex and heightened odds of being diagnosed with gonorrhoea, chlamydia, HIV, syphilis and hepatitis C virus (Glynn et al., 2018; González-Baeza et al., 2018; Pufall et al., 2018; Vu et al., 2015). In addition, chemsex has been reported to contribute to a decline in mental health, with studies reporting that people who participate in chemsex have higher odds of experiencing depression and anxiety (Berg et al., 2020; Bohn et al., 2020; Íncera-Fernández et al., 2021).

Furthermore, previous research examining the frequency of ED attendance for ketamine use has yielded contradictory findings. On one hand, investigations have found a positive correlation between escalating ketamine use and an increased rate of ED admissions compared to both other patients (Lee et al., 2011) and those who use opioids (Wei et al., 2021). Conversely, alternative studies have indicated that despite the elevated prevalence of ketamine consumption, only isolated instances of ED presentations have been documented (Chun-Jen Chen et al., 2012). It is essential to acknowledge that these aforementioned studies were confined to specific contexts and recruited in medical settings. By contrast, the current study analysed a worldwide cohort of people who use ketamine, revealing a comparatively low occurrence of EMT-seeking instances, in which less than 0.10% of intake events led people to seek medical attention. This disparity in results could potentially be attributed to the varying methodologies employed by the respective studies. Moreover, it is important to consider that respondents to the GDS are recognised for having a relatively high level of experience with drugs. This heightened familiarity may lead them to adopt precautionary measures that could potentially mitigate the need to seek EMT, in comparison to the broader population.

The findings of this study stand in contrast with substances like alcohol, which frequently lead to higher rates of medical emergencies. For instance, a study conducted in Scotland from 2016 to 2019 found that 16.2% of 536,536 ambulance callouts were alcohol-related in 2019 (Manca et al., 2021). In the Northeast of England, the prevalence of alcohol-related ED attendance was 12% in 2013, with over 31,000 (10%) alcohol-related ambulance callouts in 2012 (Martin et al., 2012; Parkinson et al., 2016). However, it is worth considering that alcohol is a legal drug, and its use is more prevalent. Additionally, an Australian study using GDS 2018 data found that 6.4% of respondents sought EMT in the past 12 months. Rates varied based on the substance used, with alcohol having the highest rate (4.30 per 100 past-year respondents who sought EMT), followed by MDMA (2.50), LSD (1.48), cannabis (0.96) and cocaine (0.67; Barratt et al., 2019). These findings suggest that, within the scope of this study, ketamine appears to carry a comparatively lower risk of triggering emergency medical situations when contrasted with other drugs.

In addition, previous studies have shown that ketamine use is rarely associated with death (Wei et al., 2021). Reports have also consistently indicated that polydrug use is prevalent among cases involving ketamine-related fatalities (Darke et al., 2021; Wood et al., 2008). The drugs commonly implicated include cannabis, alcohol, stimulants (e.g. methamphetamine) and MDMA, thus corroborating the findings of the present study, which identified that people who have used ketamine in the past year also report SDS scores that indicate probable dependence on drugs such as cannabis, cocaine and amphetamines. These findings are consistent with previous literature reporting that people who use ketamine frequently use other drugs. For instance, in the 2009–2010 GDS sample, it was found that among respondents who used ketamine, 95.5% of people reported the use of MDMA, 91.7% used cannabis and 78.7% used cocaine, among other drugs (Winstock et al., 2012).

The combination of various substances with ketamine has been linked to potential risks. For example, the lethal effects of psychostimulants such as cocaine and methamphetamine are often increased when ketamine is used (Hayase et al., 2006). This is important in the context of the findings of the present study, as respondents who used stimulants had higher odds of using ketamine, mirroring the broader pattern evident in instances of ketamine-related fatalities. Besides, this finding is consistent with previous studies that have documented that women who reported past-year cocaine use had 28 times the odds of reporting the use of ketamine (Yockey, 2023). The congruence between this study’s results and the wider body of evidence identifies implications to comprehensively address both individual health and public health systems that will be discussed in the next section.

## Implications for clinical practice

While one of the initial focuses of this study was to examine the EMT-seeking behaviour in respondents who use ketamine, the findings suggest that the frequency of respondents seeking EMT due to ketamine-related issues is relatively low compared to other drugs. For instance, a systematic review on the frequency of ED visits among adults who used illicit drugs (excluding ketamine) found that, on average, 29% of respondents sought care in the ED over a 12-month period, equivalent to an average rate of 151 visits per 100 person-years (Lewer et al., 2020). In addition, the high frequency of respondents reporting dependence on ketamine and other drugs underscores the potential for long-term harm faced by those who use ketamine (Liang et al., 2013). This prompts a re-evaluation of the weight placed on EMT provision and service delivery to manage ketamine-related concerns. Rather than solely focusing on immediate acute harms, clinicians could consider the broader implications of long-term dependence on multiple drugs, which emerged as a potential issue in this study, posing considerable risks (e.g. organ damage, cognitive impairment) for the individuals (Morley et al., 2015).

Furthermore, this study’s findings highlight that the use of other drugs in the past year, particularly stimulants, was associated with significantly increased odds of past-year ketamine use. Given the potential dangers of drug interactions and heightened health risks associated with combined substance use (Liang et al., 2013; Morgan et al., 2009; Smith et al., 2011), individuals who use ketamine need to receive information and education about the risks of drug interactions and the importance of disclosing all substances used. In this context, healthcare providers must prioritise informing individuals about the significance of minimising potential harm through informed decision-making and conducting thorough screenings that account for potential interactions among multiple drugs (Rhodes, 2009). In addition, clinicians should be aware of the potential harmful or lethal interactions between ketamine and other drugs (e.g. cocaine and methamphetamine; Hayase et al., 2006) and incorporate this knowledge into triage and treatment protocols.

Additionally, concerning individuals using ketamine within the context of parties, festivals and underground raves – particular settings that harbour a hidden and challenging-to-reach population susceptible to adverse drug-related consequences (Biolcati and Mancini, 2018) – there is a pressing need to implement targeted outreach education and harm reduction services, especially to those who use multiple substances (Fernández-Calderón et al., 2014). Such initiatives aim to empower people to self-regulate their drug use and mitigate associated harms. This is particularly crucial given the elusive nature of underground raves, where attendees may lack comprehensive insights into the potential risks they might encounter (Fernández-Calderón et al., 2014).

Moreover, the study findings indicate higher odds of ketamine use among gay men and people who use stimulants, which could be related to the use of these drugs in the context of chemsex, as the association between polysubstance use and sexual risk has been previously identified (Sewell et al., 2017). Given the well-documented links between chemsex and sexual behaviours which confer a greater risk of acquiring STIs, other communicable diseases and mental illness (Berg et al., 2020; Glynn et al., 2018; González-Baeza et al., 2018; Moreno-Gámez et al., 2022; Pufall et al., 2018; Soria, 2021; Vu et al., 2015; Wilkerson et al., 2021), healthcare practitioners should adopt a comprehensive and inclusive approach to care. This involves not only addressing substance use but also integrating sexual health education, mental health support and sexual harm reduction practices into interventions to minimise harm (Sewell et al., 2017).

Finally, in this study, a high proportion of respondents, including those who used ketamine in the past year (94.75%) and those with probable ketamine dependence (86.84%), were reluctant to seek help. People who use drugs experience different barriers when trying to access treatment. For example, the documented experiences of transgender and non-binary individuals reveal systemic discrimination linked to harmful drug use patterns, implying the existence of structural barriers to access care (Connolly et al., 2024). Additionally, experiencing stigma and prejudice contributes to the creation of a hostile and stressful environment (Hendricks and Testa, 2012; Lefevor et al., 2019; Meyer, 2003), which coupled with the presence of emotions such as embarrassment, internalised stigma and fear of discrimination can dissuade individuals and act as strong deterrents against seeking help (Clark et al., 2001; Gill et al., 2018). As a result, understanding and addressing the barriers that hinder individuals from seeking help is crucial (Connolly et al., 2020).

## Strengths and limitations of this study

The GDS recruits an international sample, allowing an in-depth understanding of stigmatised behaviours across different demographics and drug use experiences. It reaches younger and hard-to-reach populations through web-based recruitment, enriching the data with lived drug use experiences (Barratt et al., 2017). However, this study has limitations. Its cross-sectional design collects data at a single point, hindering the establishment of temporal relationships and trends. It lacks the capacity to prove causal links between correlating factors and ketamine use, and the non-probabilistic sample, limited to internet users, does not address overall population prevalence (Barratt et al., 2017; Carlson and Morrison, 2009). Furthermore, as the data presented here were collected in 2018, caution should be applied when generalising findings, and future research should consider more recent data for updated perspectives. However, the dataset remains one of the largest resources on ketamine use and behaviour changes (e.g. EMT seeking, patterns of use).

Additionally, the GDS’ non-probability-based sampling introduces volunteer bias, self-report bias, recall bias and social desirability bias (Bauhoff, 2011). Legal, social and personal concerns may impact participants’ ability to disclose their drug use. The infrequent EMT-seeking may reflect a volunteer bias, as individuals with more irregular drug use patterns and lifestyles, who may be more likely to experience acute harm, may be less likely to participate in voluntary surveys. This underrepresentation could skew our results and limit the generalisability of our findings (Johnson, 2014). While the GDS offers valuable data, its applicability to diverse populations is limited due to cultural, social and demographic differences between the general population and its respondents and the inclusion criterion requiring participants to have used at least one substance in the past 12 months. For example, in this study, the majority of respondents identified as white, limiting generalisability to ethnically and racially diverse groups.

Finally, this study relies on self-report data without the analysis of biological markers, introducing the limitation of uncertainty about the actual consumption of ketamine or other substances. For instance, recent data from an Australian drug-checking service (Olsen et al., 2022) showed that ketamine is commonly substituted with other substances, where less than half (48%) of 33 samples contained ketamine. However, the extensive scale of this study makes the inclusion of biological markers or drug testing financially and logistically challenging. Additionally, other studies have shown high fidelity between self-reported substance use and biologically confirmed results, indicating a high level of accuracy in individuals’ reporting (Bharat et al., 2023; Khalili et al., 2021).

## Conclusion

The analysis of the GDS 2018 sample identified that a high proportion of respondents reported ketamine use in the past year, 8.55% of whom met the criteria for dependence. Despite this, harm frequency remained relatively low, as few instances led to EMT. The study showed that among respondents who reported using ketamine, some also reported being dependent on other substances, notably cannabis, cocaine and amphetamines. Moreover, an association between ketamine use and stimulant consumption, as well as higher odds of use among gay men, was observed.

Considering the infrequent need for EMT, this study suggests a need to focus research efforts elsewhere. Addressing long-term health effects related to multiple drug dependencies is vital, and comprehensive screening for polydrug interactions is crucial due to the demonstrated link between ketamine use and other drugs. Finally, while the study benefits from international diversity, it has limitations such as cross-sectional design and sampling biases.

## Supplemental Material

sj-xlsx-1-jop-10.1177_02698811241273850 – Supplemental material for Ketamine use in a large global sample: Characteristics, patterns of use and emergency medical treatment Supplemental material, sj-xlsx-1-jop-10.1177_02698811241273850 for Ketamine use in a large global sample: Characteristics, patterns of use and emergency medical treatment by Karen P Barrios, Dean J Connolly, Jason A Ferris, Larissa J Maier, Monica J Barratt, Adam R Winstock, Cheneal Puljević and Gail Gilchrist in Journal of Psychopharmacology
